# Is the aspartate transaminase to platelet ratio index adequate for the assessment of liver fibrosis in patients with chronic liver disease?

**Published:** 2011-05-01

**Authors:** Hadi Parsian

**Affiliations:** 1 Department of Biochemistry and Biophysics, Babol University of Medical Sciences, Babol, IR Iran

**Keywords:** Aspartate transaminase, Liver fibrosis

Dear Editor,

I read with great interest the article by Yusuf Yilmaz et al. [1] published in a recent issue of of Hepatitis Monthly. The authors retrospectively studied 207 patients with chronic hepatitis B (CHB), 108 with chronic hepatitis C (CHC), and 140 patients with non-alcoholic fatty liver disease (NAFLD). All participants underwent liver biopsy. The stage of liver fibrosis in patients with chronic viral hepatitis was graded using the METAVIR scale. The Kleiner system for grading fibrosis was used in patients with NAFLD. As a non-invasive serum marker surrogate of liver biopsy, the APRI score was also calculated. The APRI was calculated as:

APRI=AST level/ Upper normal limit of AST/Platelet count ( 10(9) / L )×100

The authors suggested that the APRI score can be used as an appropriate non-invasive marker because it showed acceptable accuracy for the assessment of liver fibrosis in patients with CHC and NAFLD, but not in those with CHB.

We performed the same study but with a different method. The APRI score was calculated in 35 CHB patients, 14 CHC and 13 autoimmune hepatitis (AIH) patients and in 20 controls [2]. Liver histopathological parameters were evaluated by the modified Knodell score. All patients compared with the controls had higher mean ± SD APRI index (1.55 ± 1.66 vs. 0.57 ± 0.15; p < 0.001). The results showed that the AUC of the APRI (AUC = 0.840) differed significantly (p < 0.001) between the patients and controls and that patients with higher APRI score had a higher fibrosis stage, and also greater inflammation grades. It seems that we need more precise instruments for staging the liver fibrosis in addition to liver biopsy, since in liver biopsy we are dependent on the available staging systems and all these staging systems (Knodell scoring system, Kleiner system and the METAVIR scale) measure the extent of fibrosis semi-quantitatively and thus overlap between the different reported fibrosis degrees is not unlikely which limits the sensitivity and specificity of APRI score observed in the said article. Therefore, to alleviate this problem, it is proposed to measure the extent of liver fibrosis by computerized histomorphometry and also by transient elastography (fibroscan); it is better that APRI score-as non-invasive methods-be compared with these methods [3][4][5]. If the level agreement between APRI score and these techniques are acceptable, then it will be a promising method to be used instead of liver biopsy particularly in those in whom a liver biopsy is contraindicated [6][7][8]. These tests are still in their infancy, but with more extensive research, these non-invasive tests will surely assist physicians in the precise diagnosis of liver fibrosis in the future.
